# Nondestructive Testing of Local Incomplete Brazing Defect in Stainless Steel Core Panel Using Pulsed Eddy Current

**DOI:** 10.3390/ma15165689

**Published:** 2022-08-18

**Authors:** Zhiyuan Xu, Hanqing Chen, Zhongyi Qu, Changchun Zhu, Xinda Wang

**Affiliations:** School of Mechanical Engineering and Mechanics, Xiangtan University, Xiangtan 411105, China

**Keywords:** nondestructive testing, pulsed eddy current, stainless steel core panel, brazing defect

## Abstract

Stainless steel core panel is a novel structure for fast modular building, but its brazing foils are susceptible to defects due to the difficulty of precisely controlling the brazing process. An automated, nondestructive testing technique is highly desirable for quick inspection of the brazing defects buried in the stainless-steel core panel. In this paper, pulsed eddy current testing (PECT) was employed to inspect local incomplete brazing defects. Finite element simulation and experiment verification were conducted to investigate the feasibility and effectiveness of the proposed method. The peak value of the PECT signal was found to be sensitive to the presence of the defect. With the aid of an industrial robotic arm, line and two-dimensional scans were performed of the PECT probe above the panel specimen. The prefabricated incomplete brazing foil was successfully imaged as a notched ring, whose opening coincides with the physical length of the missing brazing. The proposed method shows potential to serve as an effective tool for in-line or off-line automated nondestructive testing of the brazing defects in stainless steel core panels.

## 1. Introduction

Modular building represents an innovative means of construction, in which the room-sized building components (modules) are manufactured and fitted off-site (in the factory), then transported to and installed at the construction site [1,2,3]. Compared to traditional onsite construction, the modular construction of buildings shows superiority in aspects including shorter construction periods, improved site health and safety, reduced construction waste and high-quality control at the factory. Hence, this technology is considered to have a bright application prospect in the field of construction.

The transported module is usually a framed unit with 2D panels, which means only the frame-to-frame connection is required at the construction site [3]. Since the 2D panel is the main structural element for walls and floors, many studies focused on its design and development. Lawson and Ogden [4] investigated the performance of different sheathing materials, including plasterboard, plywood, cement particleboard and steel sheeting, on a plain light steel wall panel. They found that the cement particleboard provides the greatest increase in shear resistance of the panel. Hong et al. [5] proposed a new type of lightweight sandwich panel using two skin steel plates soldered with corrugated steel plates, which was effectively used as a supplementary lateral force-resisting structure. Hickory group (a pioneer in modular construction in Australia) [6] developed a special structural unit called the Hickory Building System (HBS), which is composed of precast concrete panels. Lease Crutcher Lewis (a construction company in the US) [7] developed a composite steel-concrete-steel sandwich panel, which eliminates the need for the formwork and reinforcing bars used in reinforced concrete core construction. In the last decade, Broad Sustainable Building (BSB), a subsidiarity of Broad group in China, developed a 2D panelized construction technique for steel buildings [8,9]. The key part of this technique relies on the stainless steel (SS) core panel, which is composed of two SS plates held together with an array of thin core tubes through a patented copper brazing process. The SS core panel has an equivalent mechanical performance to the honeycomb panel used for spacecraft, but its factory fabrication costs dozens of times less than that of the honeycomb panels. To date, the panelized steel systems developed by BSB have been successfully applied to over 30 high-rise buildings, including T30 Hotel (30 stories) [10] and J57 Mini Sky Tower (57 stories) [11].

**Figure 1:**
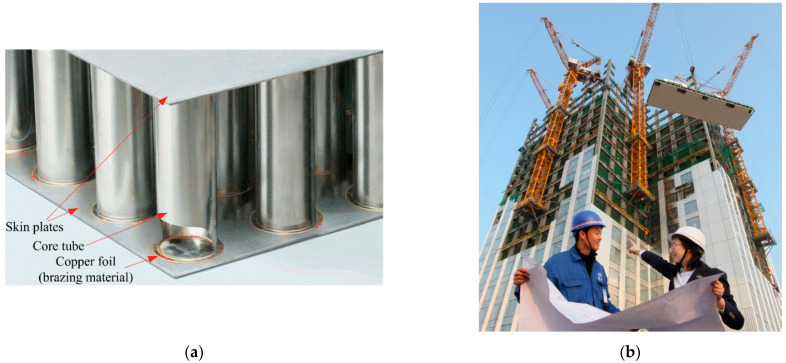
(**a**) Structure of the SS core panel and (**b**) the construction site of J57 Mini Sky Tower using the SS core panels. Reprinted/adapted with permission from Ref. [11].

Figure 1a shows the specific structure of the SS core panel. The front core tube is cut out to clearly show a brazing connection between the core tube and skin plate. The ends of the core tubes are flanged, making the brazing surface 10 times larger to solidly fuse skin plates and tubes, so that even if a tube snaps, the brazed parts do not separate. Before brazing, the panel components are stacked in an orderly manner according to the structure and packed into a dedicated hot air copper brazing oven. During the brazing process, a blower blows hot air into the oven at an extremely high speed to heat the panel components. Since the melting point of the copper foil is lower than that of the stainless steel through controlling the hot air temperature at 1100 °C, the copper foils are melted while the skin plates and core tubes are not. This forms the brazing joints and ensures that the skin plate surface can remain flat and smooth after brazing. The actual brazing process only takes a few minutes and is thus much faster and less expensive than traditional vacuum brazing processes of steel [12].

As an example, Figure 1b shows the construction site of J57 Mini Sky Tower, which uses the SS core panel as the main component. It employs some supporting techniques including the bolting assembly, triple glazing, automatic blinds and air filtration systems. The construction time was only 19 days, which is almost at a pace of three completed floors per day.

Although the thermal distortion in the copper-stainless steel brazing assembly is limited, there are many processing parameters that could induce imperfections in brazed joints, such as joint gap width, brazing temperature and interface roughness [13]. For the SS core panels, the frequent defect occurring in the brazed joints is the local incomplete brazing, which weakens the mechanical properties of the joint. As a result, there is high demand for nondestructive testing (NDT) methods, which would be applicable to this type of defect in SS core panels. In the literature, the ultrasonic-, X-ray-, and visual-techniques-based NDT methods were reportedly used to inspect the brazing defect, or similar soldering defect similars. Segreto et al. [14] used ultrasonic testing to inspect the brazed joint of two copper half-plates and found that ultrasonic testing can traverse the copper of total thickness of 17.6 mm to identify the brazing defect. Kim and Seo [15] used X-ray to inspect brazing joint defects in the heat exchanger and developed an image processing algorithm to visualize the defect. Manual visual inspection and automatic optical inspection have been widely applied to inspect solder joint defects in printed circuit boards [16,17]. However, they are only suitable for inspecting surface defects. Considering this, the BSB Company has tried ultrasonic testing and endoscopic visual inspection to inspect the incomplete brazing defect in SS core panels, but both proved insufficient for fast and accurate application. The typical size of SS core panels is 12 m long and 2 m wide and the tube density is 100 per square meter, which renders the manipulation of endoscope in visual inspection and surface preparation in ultrasonic testing time-consuming and tedious. The skin plate thickness of certain panels can be less than 1 mm, which makes the time interval between pulse echoes very short, challenging the precision of ultrasonic testing [18,19].

In this paper, pulsed eddy current testing (PECT) was employed to inspect the local incomplete brazing defect since it has been used in many successful cases to inspect subsurface and deeply buried defects. PECT inherits the merits of traditional ECT, such as being non-contact and having no need for surface preparation, which contribute to improvements in efficiency (reduction in inspection time) and excels in larger inspection depth and diverse signal features [20,21,22,23]. The local incomplete brazing defect was detected and further imaged by scanning a PECT probe above the skin plate. The feasibility and effectiveness of the proposed method were demonstrated by finite element simulation and experiment verification.

## 2. Specimen

**Figure 2:**
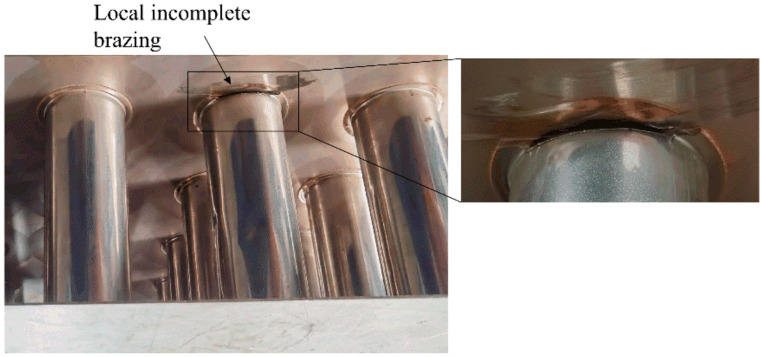
SS core panel specimen with a prefabricated local incomplete brazing defect.

Figure 2 shows the specimen provided by BSB Company. The specimen was cut from a whole SS core panel and had a length, width and height of 500 mm, 500 mm and 150 mm, respectively. The two skin plates were 1.5 mm thick and the core tubes were 51 mm in diameter and 0.5 mm in wall thickness. The tubes were flanged with a width of 5 mm and brazing connected with the skin plates. After brazing, a 0.15 mm thick brazing foil formed between the skin plate and core tube. All the tubes and plates were type 304 stainless steel, while the brazing material was annealed copper. Table 1 lists the electromagnetic properties of the specimen components required in subsequent simulations.

A local incomplete brazing defect, which is shown as the inset of Figure 2, was prefabricated at one of the core tubes. Part of the brazing foil and flanged tube wall were removed by using a handheld cutting machine. The opening accounts for about one eighth of the circular brazing foil. Still, in the opening, the foil was not completely removed, leaving some copper residues on the skin plate, as indicated by the brown stain.

## 3. Simulation

**Figure 3:**
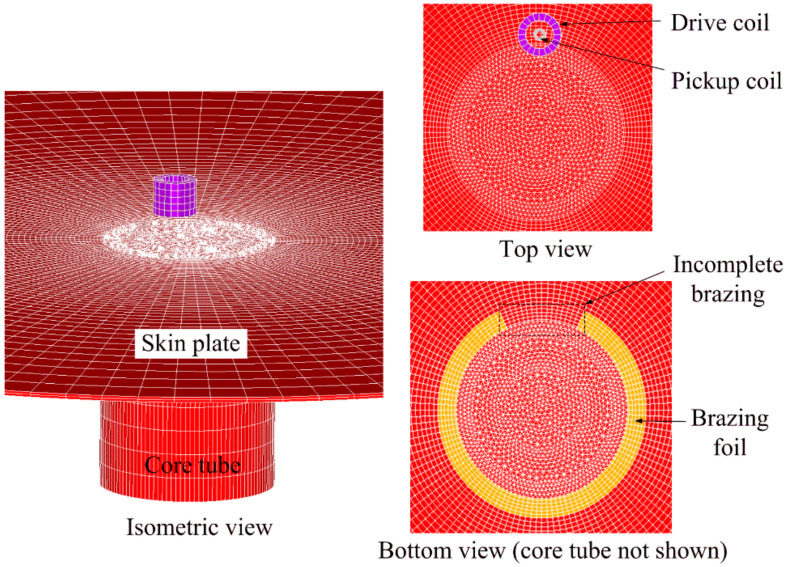
Finite element model of the SS core panel with an incomplete brazing foil.

Simulation serves as a tool for predicting the signal response and visualizing the eddy current interactions with a defect. In this section, finite element (FE) simulation is conducted to study the feasibility of PECT for inspecting incomplete brazing in SS core panels. Figure 3 shows the 3D FE model that was performed using the commercially available software ANSYS. Considering the core tubes are periodically arrayed and of the same dimension, only one core tube is modelled for simplicity. Meanwhile, since the electromagnetic field generated by the PECT probe coil was mainly concentrated in this vicinity, the skin plate near the probe was modelled while the other was not considered. The skin plate, core tube and brazing foil were assigned the electromagnetic properties listed in Table 1. The incomplete brazing defect was modelled by replacing the properties of 1/8 arc length of the brazing foil with the air properties. The probe consisted of two coaxially placed pancake coils, of which the outer and inner ones were used as the drive and pickup coils, respectively. Table 2 lists the coil parameters.

**Figure 4:**
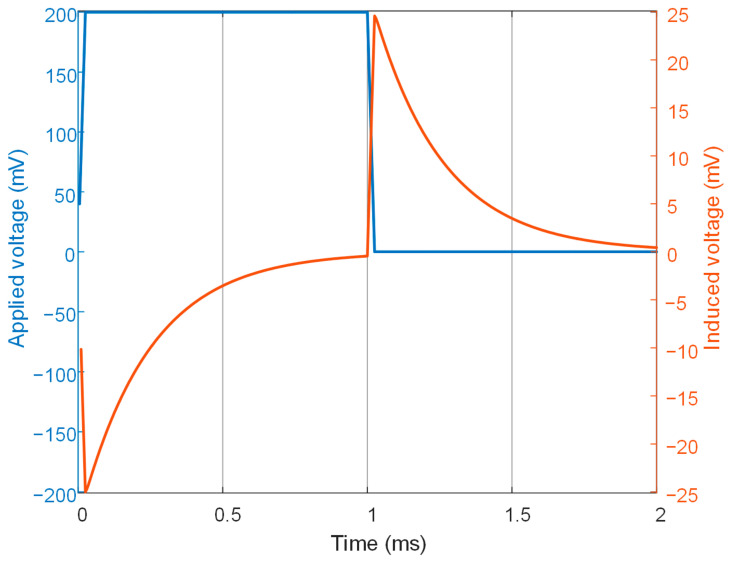
Simulated applied square-wave voltage in the drive coil and induced electromotive force in the pickup coil.

The Solid236 element with 20 nodes was selected to model all the entities. It is capable of modelling electromagnetic fields based on the ***A***-*V* formulation [23]. Neglecting the displacement current and incorporating Coulomb gauge, the governing equations are given by

(1) $$\nabla \;\times \;\frac{1}{\mu }\nabla \;\times \;A\;+\;\sigma \frac{\partial A}{\partial t}\;+\;\sigma \nabla V\;=\;J_{s}$$

(2) $$\nabla \;\cdot \;\left(\sigma \frac{\partial A}{\partial t}\;+\;\sigma \nabla V\right)\;=\;0$$

where *μ* is the magnetic permeability, *σ* the conductivity, *t* time, and ***J***_s_ the applied current density of the drive coil. ***A*** is the magnetic vector potential in the whole solution domain, whereas the electric scalar potential *V* is used only in conducting regions. As the thicknesses of the brazing foil, tube wall and skin plate were rather small compared to the dimension of the model, a meshing scheme based on the extruding mesh generation was employed to ensure a fine, even and regular mesh around the local incomplete brazing defect. In addition, mesh refinement was performed in regions where the field was changing fast. The element size for the skin plate was 0.3 mm in thickness, which is far smaller than the penetration depth of the 500 Hz eddy currents in 304 stainless steel material. As the brazing foil was ultra-thin (0.15 mm), its element size was set to 0.05 mm in thickness. The drive coil was coupled with a circuit element through which a square-wave voltage was loaded. The electromotive force (EMF) induced in the pickup coil was extracted as the PECT signal. Figure 4 shows the applied voltage in the drive coil and EMF induced in the pickup coil when the probe was oriented above the center of the local incomplete brazing, with a lift-off (the distance between probe and plate) of 1 mm. The voltage applied to the drive coil had a repetition frequency of 500 Hz, amplitude of 200 mV, duty ratio of 0.5 and rising/falling edge of 0.02 ms. The induced signal exhibited two odd-symmetric pulses, which arose at the edges of the applied voltage. The peak value of the signal was selected as the signal feature.

**Figure 5:**
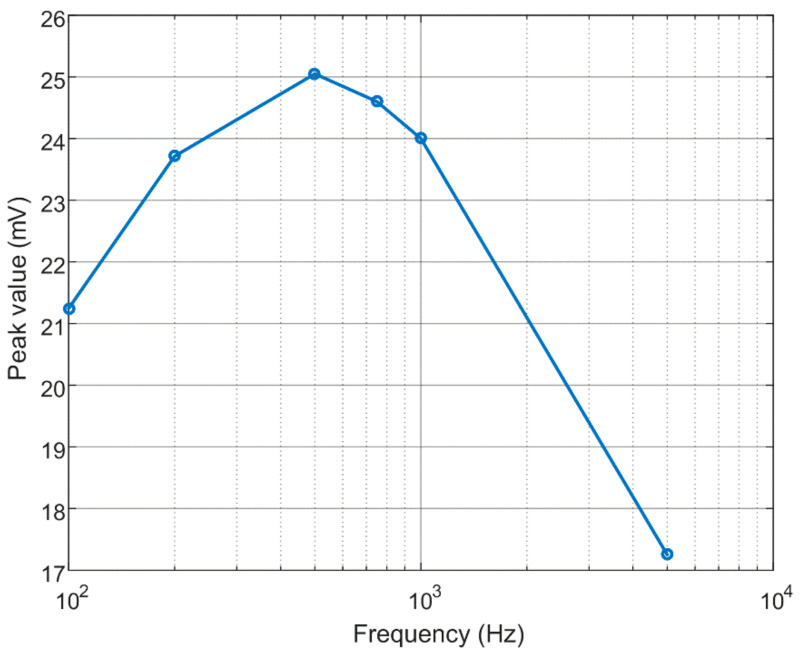
Variation in the simulated signal peak with the excitation frequency.

It is known that the inspection sensitivity has a close relationship with the eddy current penetration depth, which is inversely proportional to the frequency. The square-wave excitation can be discretized into a series of frequency harmonics and the fundamental component has the largest penetration depth. For a given conductive material, the frequency is not only concerned with the penetration depth but also the probe signal magnitude. An optimal frequency exists, at which the maximum signal is retrieved for a fixed-size defect [24]. Figure 5 shows the variation in the simulated signal peak for the drive coil excited by voltages of different repetition frequencies. The peak value reaches the maximum at the frequency of 500 Hz, as the frequency varies from 100 Hz to 5 kHz. Thus, the frequency of the applied square-wave voltage is determined to be 500 Hz.

**Figure 6:**
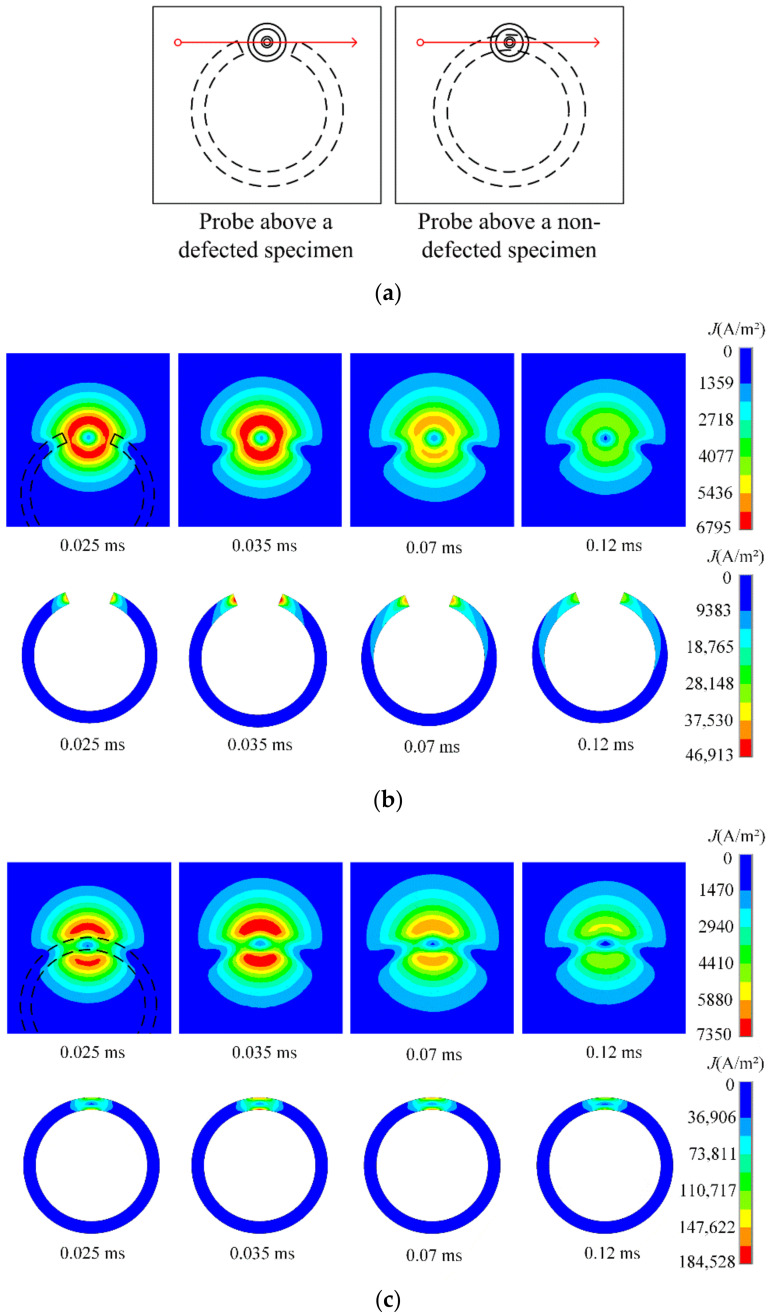
Comparison of the effect of a brazing defect on the induced eddy currents. (**a**) Diagram of probe positions relative to the brazing foil. (**b**) Distribution of eddy currents in the skin plate and brazing foil at four time points when an incomplete brazing is present in the foil. (**c**) Distribution of eddy currents in the skin plate and brazing foil at four time points when the foil is defect-free.

To examine the effect of an incomplete brazing defect on the induced eddy currents, comparative simulations were carried out between defected and non-defected specimens. Figure 6a diagrams the probe positions relative to the brazing foil in the two cases. In the model with an incomplete brazing, 1/8 arc length of the brazing foil is missing at the circumference. The FE meshes of the two models were identical to ensure a fair comparison. Figure 6b shows the distribution of eddy currents in the skin plate and brazing foil at time points of 0.025 ms, 0.035 ms, 0.07 ms and 0.12 ms, when an incomplete brazing was present in the brazing foil. Since the rising edge lasts 0.02 ms and the specified time step is 0.005 ms, the four time points correspond to steps 1, 3, 10 and 20 after the rising edge of the applied voltage, respectively. Because the brazing foil has a larger conductivity than the skin plate, the eddy current induced in the foil is much stronger than that in the skin plate, and the eddy current in the skin plate is attracted by the underlying foil, making the eddy current pattern concave in the vicinity of the foil (marked by dashed lines). The dynamic process by which the induced eddy current diffuses and decays with time can be clearly observed, especially in the brazing foil.

Figure 6c plots the distribution of eddy currents in the skin plate and brazing foil at the four time points when the foil is intact. Due to a full ring of the brazing foil, the induced eddy current is more concentrated in the foil compared to the former case. In consequence, the density of eddy current in the foil is much larger, whilst the eddy current in the skin plate shows a more concave pattern near the foil, even tending to be partitioned into two lobes. Due to this focused distribution, the diffusion of eddy current in the brazing foil is not significant.

**Figure 7:**
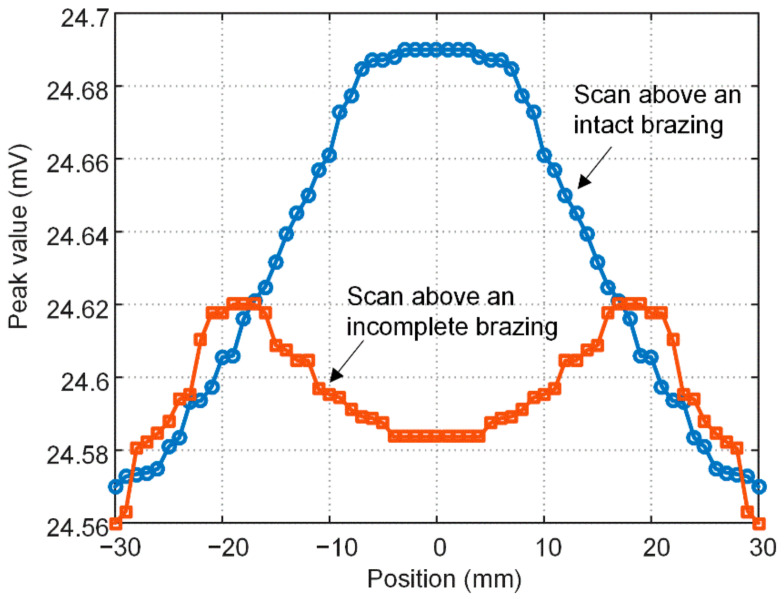
Variation in the simulated signal peak with the probe position when the brazing foil is intact and incomplete, respectively.

A line scan (B-scan) was then simulated by changing the probe position along the red line indicated in Figure 6a with a step of 1 mm. Taking the center of the circular foil as the origin x = 0, the line scan spans from x = −30 mm to x = 30 mm, yielding 61 scanning points in total. At every position, the peak value of the induced voltage in the pickup coil s acquired. Figure 7 shows the variations in peak value with the probe position in the case of an intact and an incomplete brazing foil, respectively. Because the induced eddy current in the brazing foil is stronger than that in the skin plate, the voltage signal sensed in the pickup coil increases when the probe moves towards to the brazing foil. Therefore, the variation curve bumps in the middle as the probe scans above an intact brazing, while in the scan above an incomplete brazing, the curve sinks in the brazing-missing position. The variation trend corresponds well with the aforementioned eddy current distribution and indicates the usefulness of peak value as the signal feature.

## 4. Experiment

**Figure 8:**
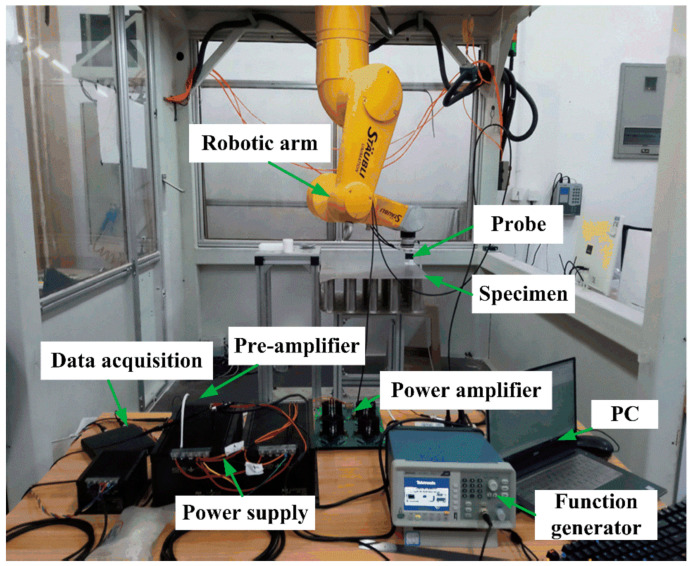
Photo of the PECT experimental set-up.

**Figure 9:**
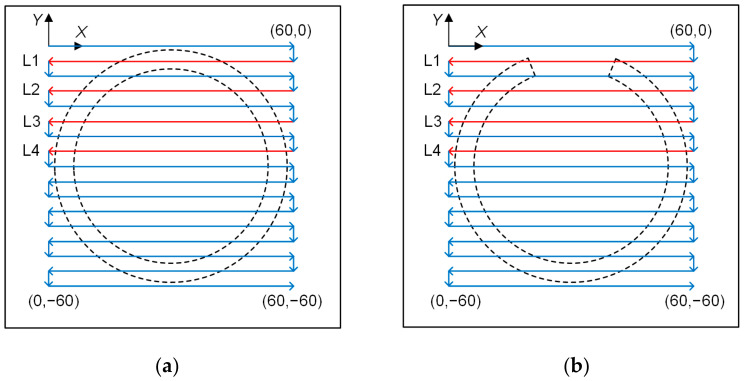
Illustration of the two-dimensional C-scan of the probe above (**a**) an intact foil and (**b**) an incomplete foil, respectively.

A lab experiment was carried out to show the effectiveness of the PECT method for inspecting the incomplete brazing defect. Figure 8 shows the experiment set-up, which mainly consists of a PECT system, a scanner and the panel specimen. Since the PECT probe was composed of a drive coil and a pickup coil, the PECT system can be described as two independent parts. A square-wave voltage generated by a function generator (AFG1022, Tektronix, Tokyo, Japan) was amplified by a homemade power amplifier. It was then fed into the probe drive coil; while the voltage signal induced in the probe pickup coil was first conditioned by a homemade pre-amplifier and sampled by a 16-bit data acquisition card (DAC) (Handyscope-HS3, TiePie Engineering, Sneek, The Netherland). Finally, it was displayed and further processed on a personal computer. The probe coils and the applied voltage used in the experiment are the same as in the simulation. An industrial robotic arm (TX2-90, Stäubli, Zürich, Switzerland) was used as the scanner. It holds the probe with a lift-off of 1 mm and controls the probe motion in the X-Y plane. At each scan position, the robotic arm sends a pulse to trigger the DAC to make a synchronous signal acquisition. To improve the signal-to-noise ratio, the acquired signals were averaged 64 times. Since there was only one defected brazing foil in the specimen and the other foils were identical, a two-dimensional scan (C-scan) was conducted above the incomplete foil with an intact foil beside it. The scan pattern is sketched in Figure 9. The scan step was 1 mm in both X and Y directions, and a total of 61 × 61 point positions were scanned. At each point, the probe stayed for 0.2 s for signal acquisition and data collection, and thus the scan time per foil was about 12 min.

**Figure 10:**
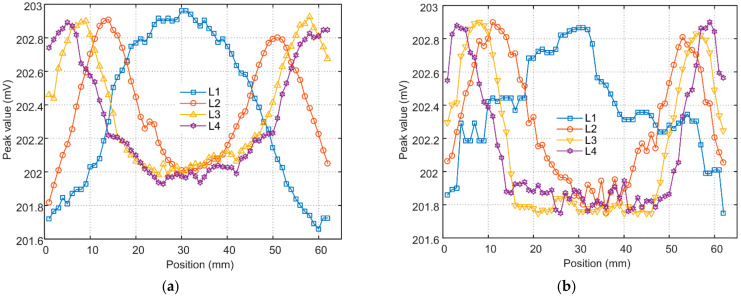
Variation in the experimental signal peak with the probe position when the brazing foil is (**a**) intact and (**b**) incomplete, respectively.

The peak values of all the acquired signals were extracted and the experimental results are represented by the variation in peak value with the scan position. Four scan lines, colored in red and denoted as L1, L2, L3 and L4 in Figure 9, were selected to show the B-scan results. Figure 10a, b shows the B-scan signals when the scanned region has an intact and an incomplete brazing foil, respectively.

Since only L1 covers the area of incomplete brazing, the middle part of the L1-scan signal rises for the intact foil, which agrees well with the simulation prediction. For the incomplete foil however, there is an abnormal signal rise from the position 20 mm to 35 mm. An examination of the defected region shows the brazing foil was not cleanly removed and some copper residues survived on the SS plate surface, as shown in the inset of Figure 2. The signals along other scan lines are in the shape of a U, and the length of the concave part increases as the scan alters from L2 to L4, which corresponds to the increase in the chord length of the brazing foil circle.

**Figure 11:**
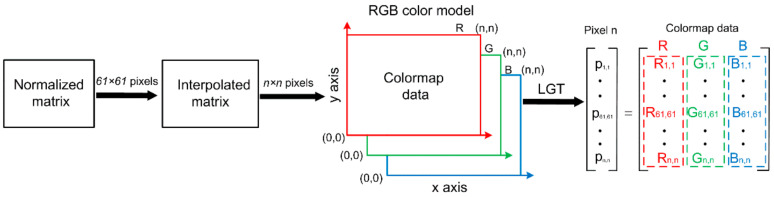
The data-processing procedure for transforming the acquired experimental signal peaks to RGB image data.

Furthermore, all the signal peak values obtained from C-scan were ordered in a 61 × 61 matrix, with their elements indexed to the scan coordinates. Ahead of imaging, the matrix data were pre-processed by using an algorithm coded in Matlab. Figure 11 illustrates the data-processing procedure. First, the row minimum and maximum values of the 61 × 61 matrix were mapped to [0,1]. Then, to improve the image resolution, a new matrix with *n* × *n* (here, *n* = 2001) elements was created by implementing a cubic interpolation among the original matrix data. Finally, the linear grayscale transformation (LGT) was performed to enhance the contrast of the image, through which the red (R), green (G) and blue (B) color components were separately transformed to the gray level and then recombined to create the C-scan color image.

**Figure 12:**
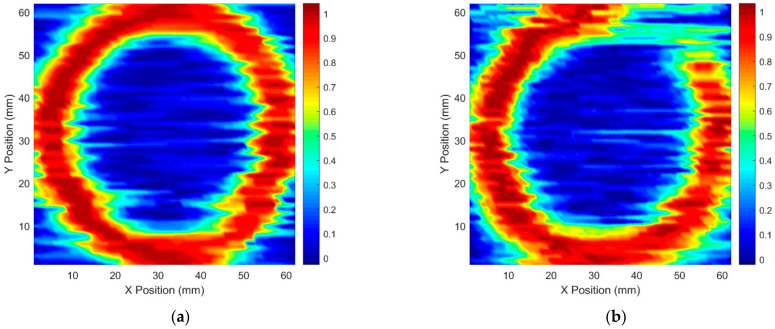
C-scan images for (**a**) an intact and (**b**) an incomplete brazing foil, respectively.

Figure 12a,b show the C-scan images when the scanned region has an intact and an incomplete brazing foil, respectively. The intact brazing foil produces a full ring in the image while the incomplete brazing foil produces a notched ring. The notch occupies about 1/8 of the ring, which matches the physical length of the incomplete brazing. The color of the notch area in the image is not that blue, which coincides with the fact that some copper residues survived in this area. The experiment results validate the effectiveness of using the PECT method for imaging the defect buried in the brazing foil of the SS core panel.

In the preliminary experiment, the detection speed is 12 min for one brazing foil, which is not that efficient. However, in actual application great improvements can be made by increasing the probe numbers and integrating the multi-channel data acquisition technique. For a 12 m long and 2 m wide SS core panel, if an array of probes is deployed and covers an entire row of brazing foils in the width direction, then the C-scan becomes a line scan along the length direction and the detection time for one row can be reduced to only 12.2 s. In this case, instead of using a robotic arm, a mechanical scanner customized according to the specific dimension of the SS core panel will be more suitable and economical. Given the above consideration, the PECT method shows promise as a fast, effective and low-cost NDT tool for the inspection of SS core panels. Table 3 summarizes the comparison of the presented method with the ultrasonic testing and visual inspection methods that have been used to inspect SS core panels.

## 5. Conclusions

This work explored the PECT technique for inspecting local incomplete brazing defects in the brazing foil of the SS core panel manufactured by the BSB Company. Finite element modeling and experiment verification were conducted to investigate the feasibility and effectiveness of the proposed method. The peak value of the PECT signal was found to be sensitive to the presence of the defect. By the aid of a robotic arm, B-scan and C-scan motions of the PECT probe above the panel specimen were performed. The prefabricated incomplete brazing foil of 1/8 arc length was successfully imaged. The proposed method is non-contact, fast and visualized and has the potential to serve as an effective tool for in-line or off-line automated nondestructive testing of the brazing defects that occur in SS core panels.

Future work will focus on the design of an arrayed probe to accommodate the requirement of highly efficient, large area scanning on the core panel. The signal processing and imaging algorithm should also be addressed further to improve the quality of C-scan images in terms of their resolution, sharpness of the edge, signal-to-noise ratio etc.

## Figures and Tables

**Table 1:** Electromagnetic properties of the specimen components.

Component	Material	Conductivity (S/m)	Relative Permeability
Skin plate	Steel, 304 Stainless	1.45 × 10^6^	1
Core tube	Steel, 304 Stainless	1.45 × 10^6^	1
Brazing foil	Copper, Annealed	5.86 × 10^7^	1

**Table 2:** Parameters of the probe coils.

Parameters	Drive Coil	Pick-Up Coil
Inner diameter (mm)	8.1	2
Outer diameter (mm)	12.5	3.5
Height (mm)	10	4
No. of turns	500	800
Wire diameter (mm)	0.25	0.05

**Table 3:** Performance comparison of NDT methods for inspecting SS core panels.

Methods	Ease of Use	Speed	Accuracy	Cost
Pulsed eddy current (presented method)	Yes, non-contact and automatic	Fast	High	Low
Ultrasonic testing	No, automatic but couplant required	Medium	High, but not applicable for skin plate thk. <1 mm	High
Visual inspection	No, manual and tedious manipulation	Slow	High, but not applicable for hidden defects	High labor costs
